# The relationship of melatonin concentration in preterm infants and adverse outcomes in the late neonatal period

**DOI:** 10.11613/BM.2023.010706

**Published:** 2022-12-15

**Authors:** Нalyna Pavlyshyn, Iryna Sarapuk, Kateryna Kozak

**Affiliations:** Department of Pediatrics No 2, I. Horbachevsky Ternopil National Medical University, Ternopil, Ukraine

**Keywords:** melatonin, preterm infants, late outcomes

## Abstract

**Introduction:**

The aim of research was to assess the melatonin concentrations in the early neonatal period as a predictor of adverse outcomes of late neonatal period in preterm infants and to estimate its optimal predictive cut-off values.

**Materials and methods:**

A total of 115 preterm infants admitted to the neonatal intensive care unit were screened for eligibility, five did not meet the criteria, six parents declined the participation. So, a total of 104 preterm infants with gestational age 25-34 weeks were included in research. The concentration of melatonin in urine was determined by the Enzyme Immunoassay method (Human Melatonin Sulfate ELISA kit, Elabscience, China). The Mann-Whitney U-test and analysis of the receiver operating characteristic (ROC) curve were used in statistical analysis.

**Results:**

Analysis of the ROC curves has revealed optimal cut-off values for urinary melatonin concentration to predict late outcomes. Melatonin concentration below 3.58 ng/ml with sensitivity of 72% can predict development of retinopathy of prematurity (ROP) (AUC = 0.73; 95% confidence intervals (CI) 0.61-0.86). Good diagnostic accuracy (AUC = 0.80; 95% CI 0.67-0.93) has been shown for bronchopulmonary dysplasia (BPD). The optimal cut-off value for melatonin concentration in BPD prediction is 3.71 ng/ml (sensitivity 80%, specificity 64%). Urinary melatonin concentration below 3.79 ng/ml can be associated with late-onset sepsis (AUC = 0.76; 95% CI 0.64-0.87; sensitivity 72%; specificity 62%). There were no significant associations between melatonin concentration and necrotizing enterocolitis (P = 0.912).

**Conclusion:**

Urinary melatonin concentration below the certain cut-off values in the early neonatal period may serve as one of the predictors of adverse outcomes such as BPD, ROP, and late-onset sepsis in the late neonatal period in preterm infants.

## Introduction

Today, the number of preterm infants is increasing all over the world. According to the World Heath Organisation (WHO), about 15 million children are born preterm every year, that is more than one child out of ten. The preterm birth rate ranges from 5 to 18% of all births in 184 countries, with more than 60% of preterm births occurring in Africa and South Asia (*1*).

Preterm newborns are at risk of neonatal disorders related to immaturity. Thus, they often suffer from respiratory distress syndrome (RDS), necrotizing enterocolitis (NEC), intraventricular haemorrhage (IVH), bronchopulmonary dysplasia (BPD), retinopathy of prematurity (ROP), early- and late-onset infections (*2*). Free radical-mediated tissue damage due to oxidative stress is a common factor in the pathogenesis of these diseases (*3*, *4*). Oxidative stress is defined as an imbalance between the production of pro-oxidant substances and the protective antioxidant system, with the predominance of pro-oxidants, which can lead to damage (*5*). Hyperoxia has a toxic effect on various organs and systems, and especially on the preterm infant’s organism (*6*).

Preterm neonates are particularly sensitive to oxidative stress due to an imbalance between increased reactive oxygen species (ROS) production because of often additional oxygen supply and insufficient antioxidant capacity (*7*-*9*). Hyperalgesia as a result of frequent and numerous painful procedures which preterm neonates are exposed to in the intensive care units leads to the additional free radicals production (*10*).

Endogenous melatonin is a powerful antioxidant and scavenger of free radicals (*3*). Melatonin and its metabolites effectively interact with various ROS, as well as with organic radicals, increase the activity of antioxidant enzymes and reduce the activity of pro-oxidant enzymes (*9*). It also has anti-inflammatory, anti-apoptotic, anti-excitotoxic, and immunomodulatory properties (*5*, *9*).

Recent studies suggest a protective role for melatonin from oxidative damage, particularly in newborns. Thus, melatonin has positive antioxidant influence in chronic lung diseases, perinatal brain damage, NEC, ROP, and sepsis (*6*, *11*-*13*).

The aim of the research was to assess the melatonin concentrations in the early neonatal period as a predictor of adverse outcomes of the late neonatal period in preterm infants and to estimate it optimal predictive cut-off values.

## Materials and methods

### Study design

This is a prospective, longitudinal study. A total of 115 preterm infants who were admitted to the neonatal intensive care unit of the Regional Perinatal Center were consecutively screened for eligibility, and five did not meet the criteria for this study because of exclusion criteria, six parents (5.2%) declined the participation and did not sign the informed consent. So, a total of 104 preterm infants with gestational age (GA) 25-34 weeks were included in this research.

Inclusion criteria: prematurity (GA 25 0/7 – 33 6/7 weeks) and obtained parental consent. Exclusion criteria: chromosomal disorders and congenital malformations.

The WHO defines extremely preterm infants as those born at < 28 weeks of gestation, very preterm as those born at 28–32 weeks of gestation, and moderate preterm 32 weeks to < 34 weeks. Small for gestational age (SGA) was defined as a birth weight of < 10^th^ percentile for gender and GA (*14*).

### Methods

Urine was collected during 2-3 hours from 1 pm to 4 pm for all infants. All the day and night time infants were in the same light conditions – in the incubators that were covered with dark covers to protect them from the bright light of the intensive care unit. Urine was collected using cotton sponges, and then was extracted by centrifugation for 2 minutes. After extraction, samples were centrifuged at 1000xg at 2-8°C for 20 min and stored at – 80°C. Quantitative determination of melatonin sulfate in the urine was performed by the Enzyme Immunoassay method (Human Melatonin Sulfate ELISA kit, Elabscience, Wuhan, China). The inter-individual coefficient of variation (CV) was 5.54%.

The study was conducted according to the World Medical Association’s Helsinki Declaration. The research design was approved by the University Ethical Committee, protocol No 60 from 1 September 2020. All parents of eligible infants have signed the informed consent.

### Statistical analysis

Statistical analysis was performed by the Statistica 13.0 program (StatSoft Inc., Tulsa, USA). All data sets have been tested for normality by the Shapiro-Wilk’s test, which showed the necessity to use nonparametrics statistical methods. Quantitative data are presented as the median and interquartile range (IQR; 25th to 75th percentiles); qualitative data – as absolute and relative frequencies. The Mann-Whitney U-test (for two independent groups) was used to compare numerical data. A probability level of less than 0.05 was considered as statistically significant. Analysis of the receiver operating characteristic (ROC) curve was done to determine the optimal cut-off values in adverse outcomes prediction. Classifier performance was assessed based on the area under the ROC curve (AUC) analysis, and 95% confidence intervals (CI) for AUC was calculated. Sensitivity and specificity for diagnostic tests were evaluated.

## Results

A total of 104 preterm infants born between 25 0/7 and 33 6/7 gestational weeks were enrolled in the research. There were 68 (65%) extremely and very preterm infants and 36 (35%) moderate preterm infants. The median and min-max of GA was 32.0 (25.0-34.0) weeks; median birth weight 1650.0 (1300.0-1900.0) g. Ten infants (10%) were born small for gestational age. There were 55 (53%) males and 49 (47%) females. Characteristics of infants in the early neonatal period are presented in Table 1. Forty-four infants had adverse outcomes in the late neonatal period: 18 neonates (17.3%) had late-onset sepsis, 18 (17%) – ROP, 10 (9.6%) – BPD, and 20 (19%) – NEC, 16 (15%) – had a combination of 2 or 3 diseases. All infants with late outcomes were included in the study group. Sixty preterm infants (57.7%) did not have late outcomes, thus they were considered as a control group.

**Table 1:** Characteristics of infants in the early and late neonatal period

**Characteristics of infants in the early neonatal period**	**Statistical indicators**	**Study group,** **N = 104**
Apgar score at the 1^st^ min	Median (IQR)	7.0 (6.0-7.0)
Apgar score at the 5^th^ min	Median (IQR)	7.0 (7.0-7.0)
Primary resuscitation	N (%)	52 (50%)
Surfactant replacement therapy	N (%)	32 (31%)
Respiratory distress syndrome	N (%)	79 (76%)
Early-onset infection	N (%)	30 (29%)
Severe neurological disorders	N (%)	22 (21%)
Intraventricular haemorrhage	N (%)	25 (24%)
Mechanical ventilation	N (%)	27 (26%)
Continuous positive airway pressure-therapy	N (%)	64 (62%)
IQR – interquartile range.		

Median urine melatonin concentration in the study group of preterm newborns was 3.15 (1.95-4.89) ng/ml, in the control group 4.65 (2.50-6.77) ng/ml, P = 0.021. Urine melatonin concentrations in neonates with different adverse late outcomes are presented in Table 2.

**Table 2:** Melatonin concentration in preterm newborns depending on the late outcomes

	**N**	**Melatonin, ng/ml**	**P value**
Late-onset sepsis	18	2.73 (0.77-4.00)	0.001
Control group	60	4.65 (2.50-6.77)	
ROP	18	2.44 (1.69-4.80)	0.003
Control group	60	4.65 (2.50-6.77)	
BPD	10	2.22 (1.09-3.40)	0.003
Control group	60	4.65 (2.50-6.77)	
Necrotizing enterocolitis	20	3.43 (2.77-8.07)	0.916
Control group	60	4.65 (2.50-6.77)	
Melatonin concentrations are presented as median and interquartile range. P values < 0.05 were considered statistically significant. ROP - retinopathy of prematurity. BPD - bronchopulmonary dysplasia.			

**Figure 1:**
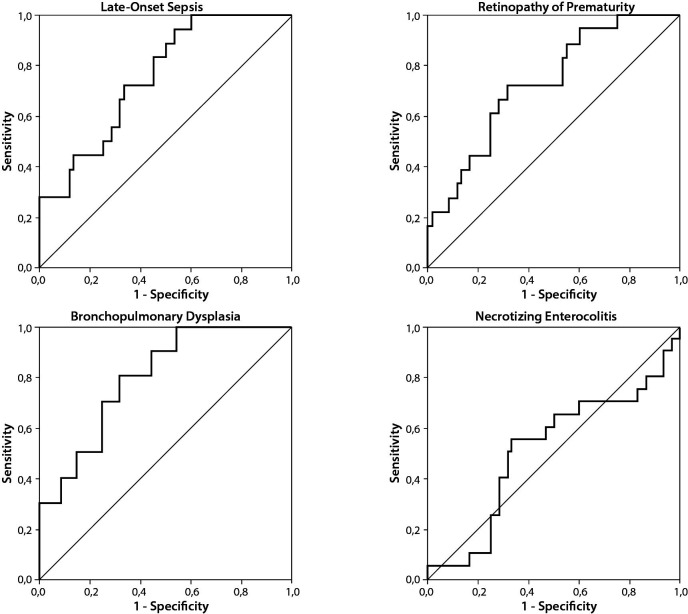
ROC curve analysis for assessing urinary melatonin concentrations in the early neonatal period in predicting the adverse outcomes in the late neonatal period

Analysis of ROC curves has revealed optimal cut-off values for urinary melatonin concentration to predict development of late adverse outcomes. Decrease of the urinary melatonin concentration below 3.58 ng/ml with sensitivity of 72% can predict development of retinopathy of prematurity (AUC = 0.73; 95% CI 0.61-0.86). Good diagnostic accuracy (AUC = 0.80; 95% CI 0.67-0.93) has been shown for bronchopulmonary dysplasia. The optimal cut-off value for urinary melatonin concentration in case of bronchopulmonary dysplasia prediction is 3.71 ng/ml (sensitivity 80% and specificity 64%). Urinary melatonin concentration below 3.79 ng/ml can be associated with late-onset sepsis in preterm newborns (AUC = 0.76; 95% CI 0.64-0.87; sensitivity 72%; specificity 62%). Nevertheless, the same (3.79 ng/ml) urinary melatonin concentration has poor diagnostic ability in the prediction of NEC (AUC = 0.51; 95% CI 0.36-0.66; P = 0.912). (Table 3, Figure 1).

**Table 3:** Melatonin concentration and late outcomes prediction in preterm newborns

	**Late-Onset sepsis**	**Retinopathy of prematurity**	**Bronchopulmonary dysplasia**	**Necrotizing enterocolitis**
AUC (95% CI)	0.76 (0.64−0.87)	0.73 (0.61−0.86)	0.80 (0.67−0.93)	0.50 (0.36−0.66)
P value	0.001	0.003	0.003	0.912
Cut-off point	3.79	3.58	3.71	3.79
Sensitivity (%)	72	72	80	55
Specificity (%)	62	65	64	62
P values < 0.05 were considered statistically significant. AUC – area under curve. CI – confidence intervals.				

## Discussion

Our study showed the predictive cut-off values of melatonin concentrations in the early neonatal period for adverse outcomes (BPD, ROP and late-onset sepsis) in late neonatal period. We found that urinary melatonin concentration below 3.71 ng/ml in the early neonatal period was associated with the BPD development. This data indicates that in case of insufficient melatonin concentration, the immature organism of preterm infants is not able to provide full antioxidant and anti-inflammatory protection to prevent the development of lung tissue damage with chronic lung disease occurrence. Bronchopulmonary dysplasia is one of the specific diseases of prematurity that are called «free radical diseases of prematurity» due to the oxidative stress as the main component in their pathogenesis (*4*, *15*). Oxidative stress with increased free radicals production cause tissue damage, and thus contribute to the BPD development (*9*). Also an inflammatory reaction develops in response to lung tissue damage by oxygen, and elevated levels of pro-inflammatory cytokines are found in higher concentrations in children who develop chronic lung diseases (*16*, *17*). Some authors also suggest that lung damage in BPD is associated not only with the influence of active oxygen radicals, but with a decrease in antioxidant activity and the insufficient protective functions in preterm neonates (*12*, *18*). As shown in our results, the sensitivity and specificity of the ROC curve analysis were quite high (80% and 64%, respectively), indicating that low urinary melatonin concentrations in the early neonatal period could predict the development of BPD with high reliability.

Authors suggest that melatonin, possessing powerful antioxidant and anti-inflammatory properties, participates in the protection of immature lungs from oxidative damage (*5*, *19*). Data on the assessment of endogenous melatonin and its effectiveness in the prevention of BPD are limited, but some authors have investigated the efficacy of exogenous melatonin in RDS in premature infants. These studies have shown that melatonin reduced oxidative stress and serum pro-inflammatory interleukins in neonates with RDS, decreased pro-inflammatory cytokines in tracheobronchial aspirate, and improved outcomes due to its antioxidant effects, thus have been reducing the risk of BPD (*16*, *19*, *20*). In addition, Choi *et al.* found that melatonin promoted the development of lung architecture and protected the lungs during critical alveolarization processes after preterm birth (*21*).

Retinopathy of prematurity is also one of the free radical diseases, and oxidative stress contributes to it pathogenesis. The use of supplemental oxygen, high oxygen concentrations, and long-term mechanical ventilation are the most frequently risk factors for severe and treatment-requiring ROP (*22*, *23*). Our research showed that the melatonin concentration below 3.58 ng/ml was associated with the occurrence of ROP, which confirms that an insufficient concentration of melatonin as an antioxidant leads to an increased risk of free radical diseases developing. Sensitivity and specificity of 72% and 65%, respectively, suggests that melatonin can be a reliable predictor of the ROP development.

Authors indicate that exogenous melatonin therapy has positive impact on the visual functions of preterm infants (*9*). It has been proven that melatonin modulates the activity of retinal neurons by activating different subtypes of melatonin receptors present on the retinal neurons (*24*).

Melatonin acts not only as a free radical scavenger and antioxidant, but also has potent anti-inflammatory properties, that are very important for preterm infants. Pathogenesis of different diseases, particularly neonatal sepsis include the combination of inflammation with oxidative stress (*4*, *25*). Recent studies revealed the involvement of free radicals in the development of neonatal sepsis and its complications (*5*). Our results also showed that melatonin concentration below 3.79 ng/ml was associated with the occurrence of late-onset neonatal sepsis (sensitivity and specificity 72% and 62%, respectively).

Studies with exogenous melatonin administration to neonates with sepsis have shown significant clinical efficacy, oxidative stress reduction, decrease in the pro-inflammatory serum markers concentration (*8*, *13*). Melatonin has also been shown to improve survival in neonates with sepsis and septic shock and may reduce ventilator-associated lung injury in preterm infants (*19*, *26*). The effectiveness of exogenously administered melatonin indicates that the amount of endogenous melatonin in the preterm infants’ organism is insufficient to effectively counteract severe pro-oxidant, pro-inflammatory processes that occur during the neonatal sepsis.

There were no significant associations between melatonin concentration and NEC (AUC = 0.51; 95% CI 0.36-0.66; P = 0.912). Data on the assessment of endogenous melatonin at NEC are limited, but there are experimental studies about the potential therapeutic effect of exogenous melatonin supplementation to modulate NEC pathogenesis (*27*, *28*).

Determination of the melatonin concentration in the early neonatal period as a reliable predictor of the BPD, ROP and late-onset neonatal sepsis development will allow to classify certain categories of newborns who need some interventions to increase the melatonin secretion to prevent the development of these diseases with their negative outcomes.

The strength of this study is that this is the first national research of melatonin as a predictor of neonatal diseases in preterm infants. The main limitation of our study is the small sample size of preterm infants, especially extremely preterm newborns. Also, the fact that many other factors that contribute to adverse late outcomes have not been tested in these subjects.

In conclusion, this prospective study showed that a low urinary melatonin concentrations in the early neonatal period may serve as one of the reliable predictors of adverse neonatal outcomes such as BPD, ROP and late-onset sepsis in the late neonatal period in preterm infants.

## Data Availability

The data generated and analysed in the presented study are available from the corresponding author on request.
